# Supplementary figures and images for: Thermosensory behaviors of the free-living life stages of Strongyloides species support parasitism in tropical environments
Source: PLoS Negl Trop Dis. 2024 Dec 17;18(12):e0012529. doi: 10.1371/journal.pntd.0012529 (PMC11687888; doi:10.1371/journal.pntd.0012529)

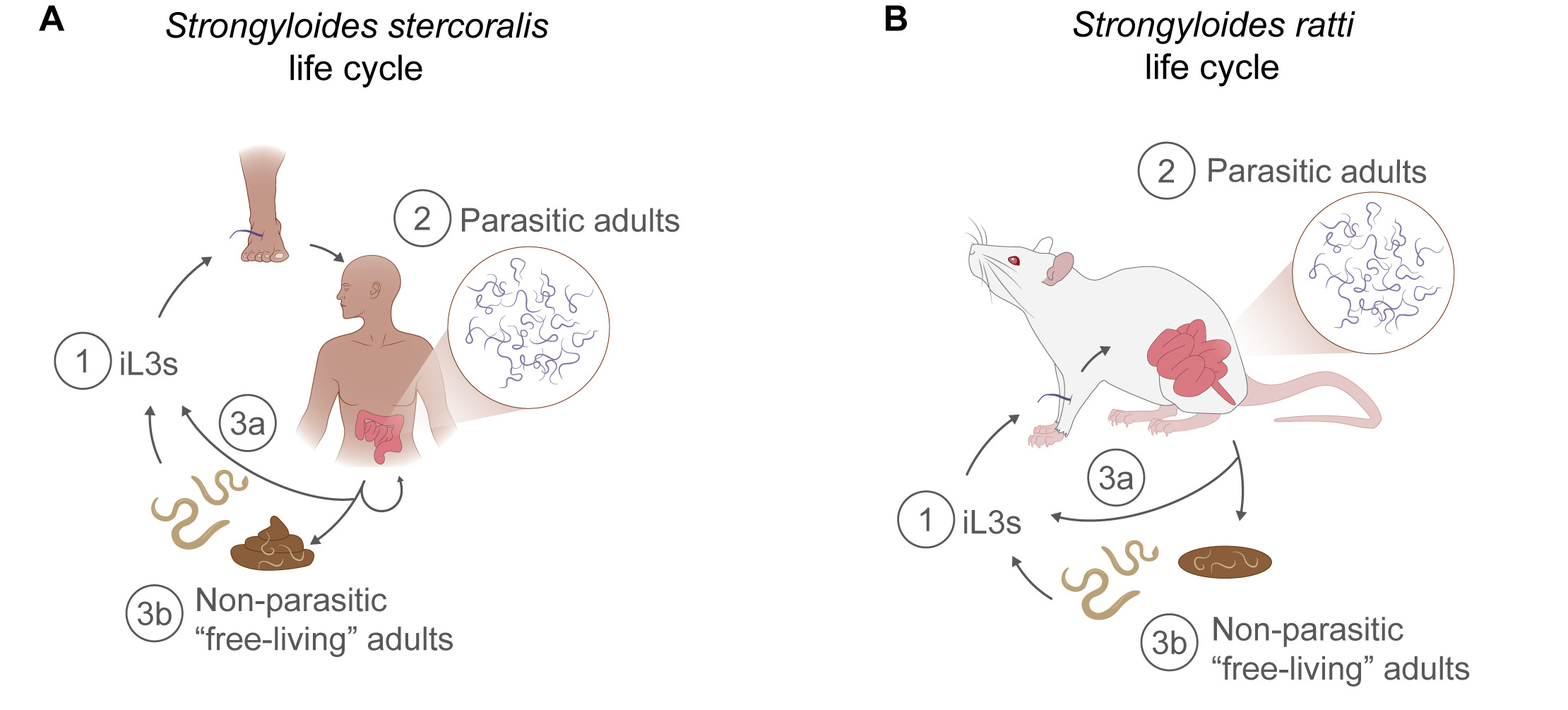

Supplement: S1 Fig — A) The life cycle of S. stercoralis in a human host [8]. 1) Infection starts when soil-dwelling infective third-stage larvae (iL3s) locate a host and skin penetrate. 2) Inside the host, iL3s migrate to the small intestine and develop into reproductively active parasitic females. The eggs and larvae of parasitic females are voided from the host in feces, where they can either develop directly into iL3s (3a) or into non-parasitic “free-living” adults that engage in sexual reproduction (3b). The offspring of the free-living adults all develop into iL3s. In S. stercoralis, autoinfection can occur when eggs hatch within the host and develop into autoinfective larvae. B) The life cycle of S. ratti in a rat host [19]. The stages of infection are similar to S. stercoralis, with the following differences: a) the progeny of parasitic females leave the host exclusively as unhatched eggs; b) the lack of precocious hatching precludes autoinfection of original hosts. (TIF) [file pntd.0012529.s001.tif]

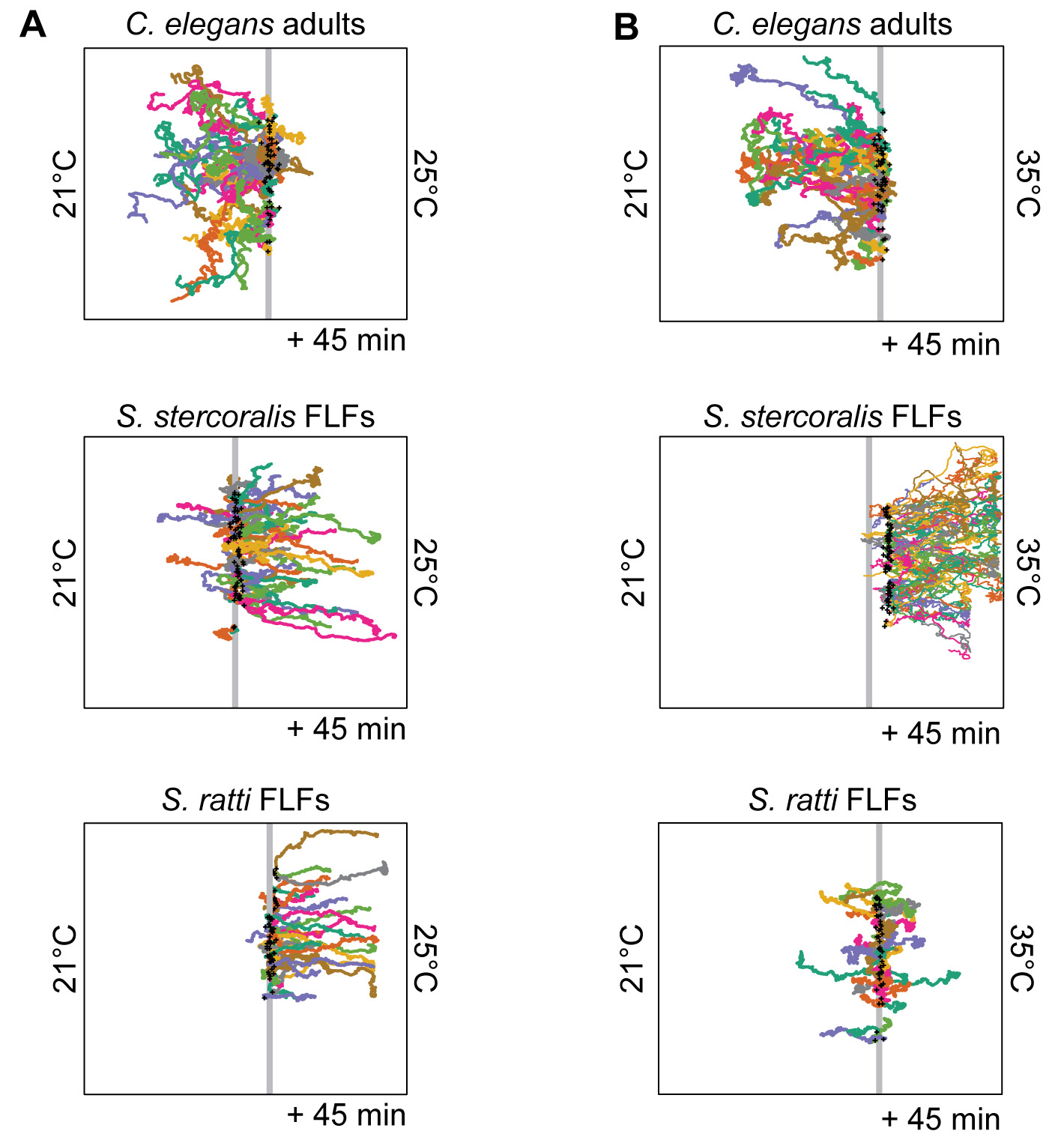

Supplement: S2 Fig — All tracks of C. elegans adult hermaphrodites, S. stercoralis free-living females (FLFs), and S. ratti FLFs migrating in either a ~21–25°C gradient (A) or a ~21–35°C gradient (B). For panel A, cultivation temperature (TC) = 20°C and starting temperature (Tstart) = 23°C (grey line). A subset of the tracks plotted here is also shown in Fig 1. For panel B, TC = 23°C and starting temperature (Tstart) = 30°C (grey line). A subset of the tracks plotted here is also shown in Fig 2. For the ~21–25°C gradient, n = 54 worms for C. elegans hermaphrodites (5 assays across 4 days), n = 76 worms for S. stercoralis free-living females (7 assays across 5 days), n = 59 worms for S. ratti FLFs (6 assays across 4 days). For the ~21–35°C gradient, n = 50 worms for C. elegans hermaphrodites (5 assays across 3 days), n = 65 worms for S. stercoralis FLFs (5 assays across 3 days), n = 47 worms for S. ratti FLFs (6 assays across 4 days). (TIF) [file pntd.0012529.s002.tif]

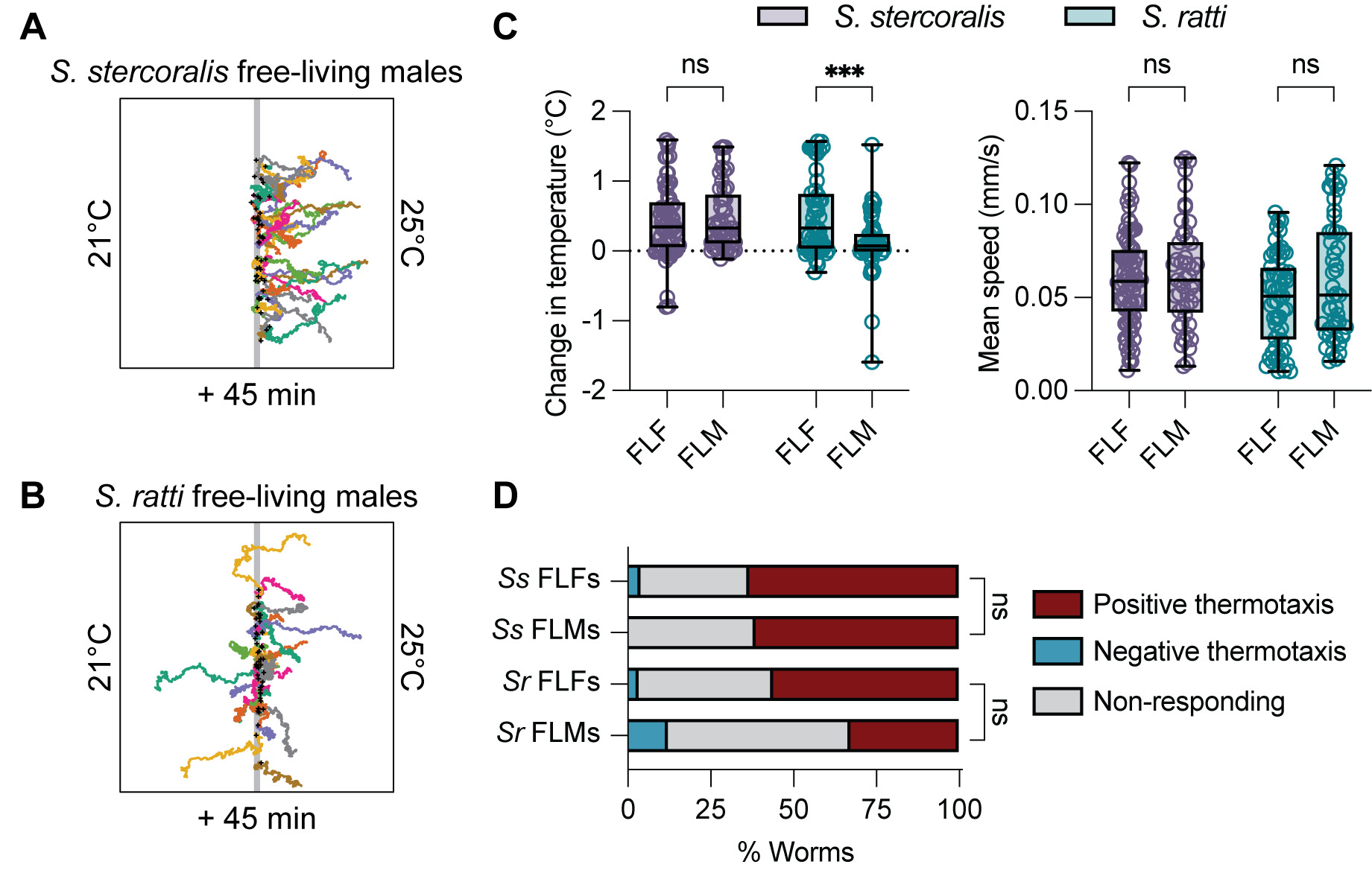

Supplement: S3 Fig — A) Individual tracks of S. stercoralis free-living males responding to a ~21–25°C temperature gradient (Tstart = 23°C). TC = 20°C. Each colored line is the track of an individual S. stercoralis male’s path throughout the 45-minute assay. Black crosses represent the starting positions of each worm. The grey line represents Tstart = 23°C. B) Individual tracks of S. ratti free-living males responding to a temperature gradient from ~21°C to 25°C when placed at 23°C. Each colored line is the track of an individual S. stercoralis male’s path throughout the 45-minute assay. Black crosses represent the starting positions of each worm. The grey bar represents a 1°C-wide starting zone of the assay and is centered on 23°C. C) Quantification of the change in temperature (left) and mean speed (right) for free-living males (FLMs) vs. free-living females (FLFs) of S. stercoralis (purple) or S. ratti (teal). Icons indicate responses of individual worms, boxes show medians and interquartile ranges, and whiskers show min and max values. S. stercoralis FLMs and FLFs migrated similarly in the temperature gradient; S. ratti FLMs showed reduced positive thermotaxis relative to S. ratti FLFs. The mean speed of worms in the gradient was not significant between the sexes. n = 76 worms for S. stercoralis FLFs (7 assays over 5 days), n = 49 worms for S. stercoralis FLMs (5 assays over 3 days), n = 59 for S. ratti FLFs (6 assays over 4 days), and n = 49 for S. ratti FLMs (5 assays over 4 days). Icons indicate responses of individual worms, boxes show medians and interquartile ranges, and whiskers show min and max values. ns = not significant, ***p<0.001, two-way ANOVA with Šídák’s multiple comparisons test. D) Categorical distribution of thermotaxis behaviors in a ~21–25°C gradient across species. Individuals were considered to have engaged in positive or negative thermotaxis if their position at the end of the assay was outside of a 1 cm neutral exclusion zone centered on the starting positio [file pntd.0012529.s003.tif]

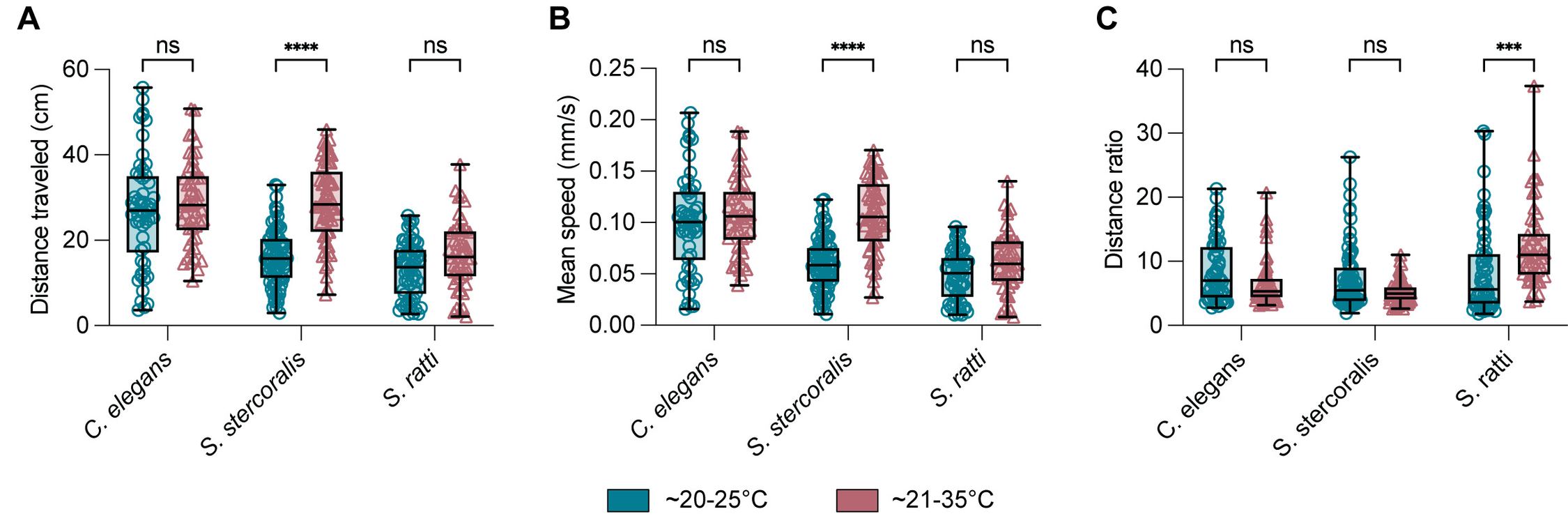

Supplement: S4 Fig — Quantification of the total distance traveled (A), mean speed (B), and distance ratio (C) of worms placed in either the ~21–25°C gradient (teal) or the ~21–35°C gradient (rose). Distance traveled was calculated for each worm as the summation of Euclidean distances between sequential measured X/Y coordinates. Distance ratio was calculated for each worm by dividing the total distance traveled by the maximum Euclidean displacement from its starting position. A larger distance ratio represents a more circuitous path; a distance ratio of 1 is a completely straight path. For all panels, icons indicate responses of individual worms, boxes show medians and interquartile ranges, and whiskers show min and max values. ns = not significant, ***p = 0.0001, ****p<0.0001, two-way ANOVA with Sidak’s multiple comparisons test. For the ~21–25°C gradient, n = 54 worms for C. elegans hermaphrodites (5 assays across 4 days), n = 76 worms for S. stercoralis free-living females (7 assays across 5 days), n = 59 worms for S. ratti FLFs (6 assays across 4 days). For the ~21–25°C gradient, n = 50 worms for C. elegans hermaphrodites (5 assays across 3 days), n = 65 worms for S. stercoralis FLFs (5 assays across 3 days), n = 47 worms for S. ratti FLFs (6 assays across 4 days). (TIF) [file pntd.0012529.s004.tif]

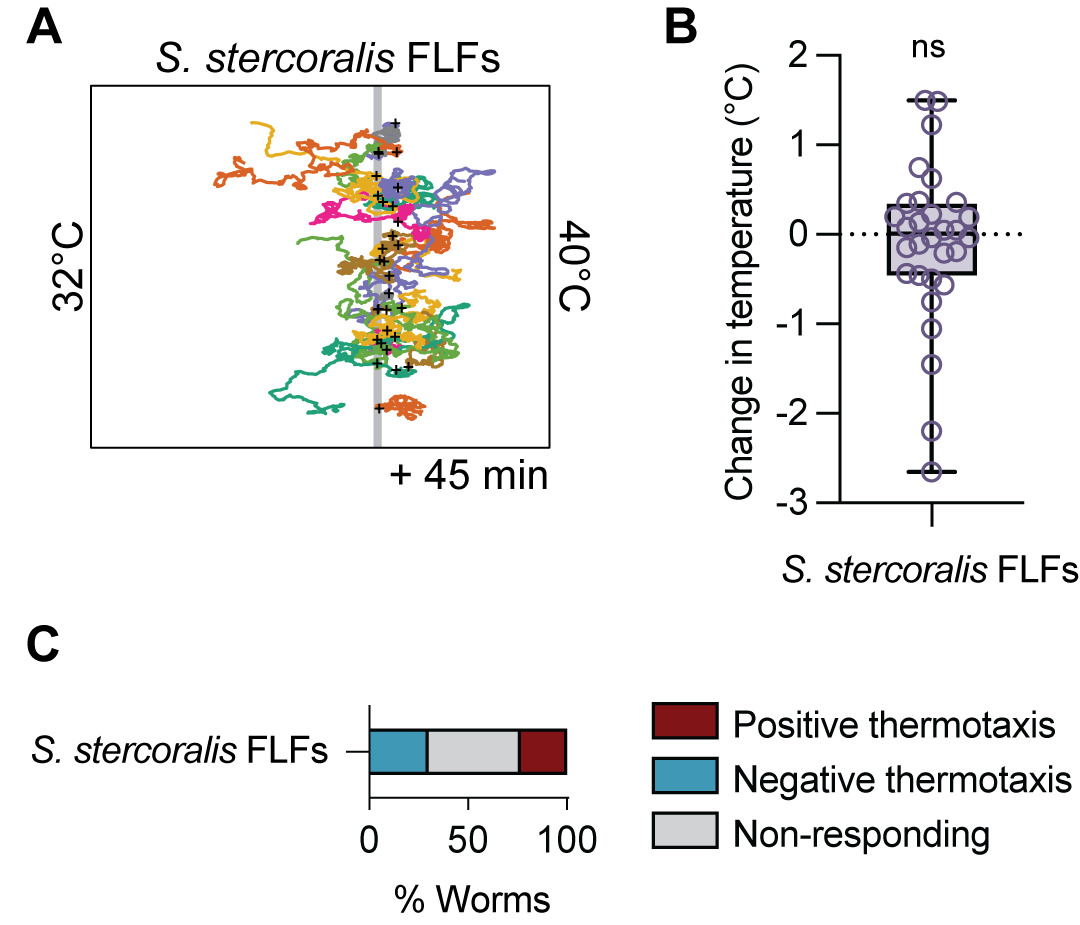

Supplement: S5 Fig — A) Individual tracks of S. stercoralis free-living females responding to a ~32–40°C temperature gradient (Tstart = 38°C). TC = 23°C. Each colored line is the trajectory of an individual worm during the 45-minute assay. Black crosses represent the starting positions of each worm. The grey line represents Tstart = 38°C. B) Quantification of the change in temperature for S. stercoralis free-living females. Values are final temperature–starting temperature for each worm. Icons indicate responses of individual worms; boxes show medians and interquartile ranges; whiskers show min and max values. ns = not significantly different from a hypothetical value of 0, Wilcoxon signed-rank test. C) Categorical distribution of S. stercoralis thermotaxis behavior in a ~32–40°C gradient. Individuals were considered to have engaged in positive or negative thermotaxis if their position at the end of the assay was outside of a 1 cm neutral exclusion zone centered on the starting position of each individual worm. Individuals that finished the assay within this zone were considered non-responding. n (negative/non-responding/positive) = 9/14/7. (TIF) [file pntd.0012529.s005.tif]

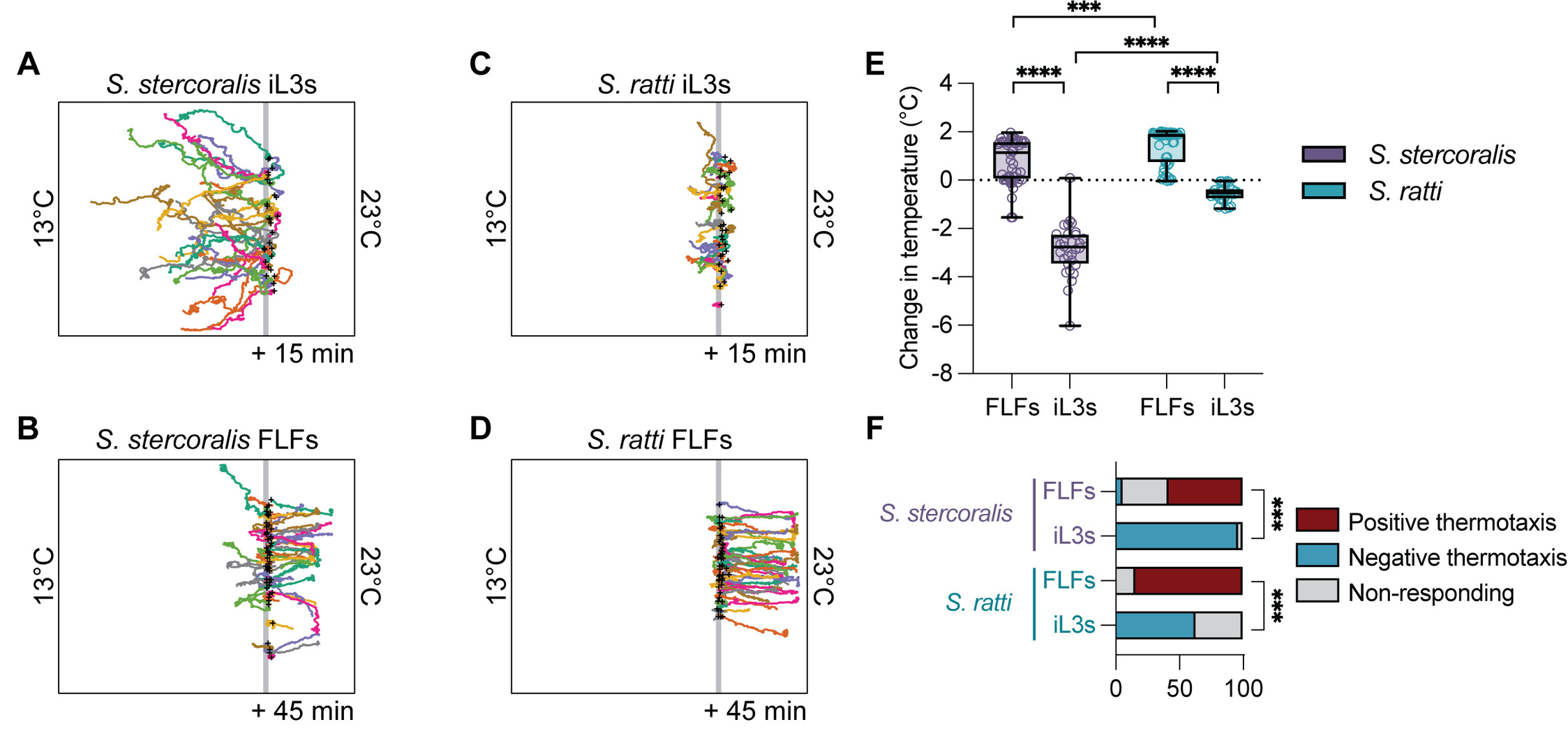

Supplement: S6 Fig — A-D) Tracks of S. stercoralis iL3s (A), S. stercoralis FLFs (B), S. ratti iL3s (C), and S. ratti FLFs (D) in a ~13–23°C gradient (Tstart = 20°C, TC = 23°C). Each colored line is an individual worm’s path throughout the 15-minute assay. Black crosses represent the starting positions of each worm. The grey line represents Tstart = 20°C. Assay duration: 45 minutes (adults) or 15 minutes (iL3s). E) Quantification of the change in temperature experienced by worms in the ~13–23°C gradient. Icons indicate responses of individual worms, boxes show medians and interquartile ranges, and whiskers show min and max values. n = 30–50 worms from 3–5 assays. ***p<0.001, ****p<0.0001, two-way ANOVA with Tukey’s multiple comparisons test. F) Categorical distribution of thermotaxis behaviors in a ~13°C-23°C gradient across species. Individuals were considered to have engaged in positive or negative thermotaxis if their position at the end of the assay was above or below 0.5°C of their starting position, respectively. Individuals that finished the assay within 0.5°C above or below Tstart were considered non-responding. ***p<0.001, Fisher’s exact test with Bonferroni-Dunn correction for multiple tests. (TIF) [file pntd.0012529.s006.tif]

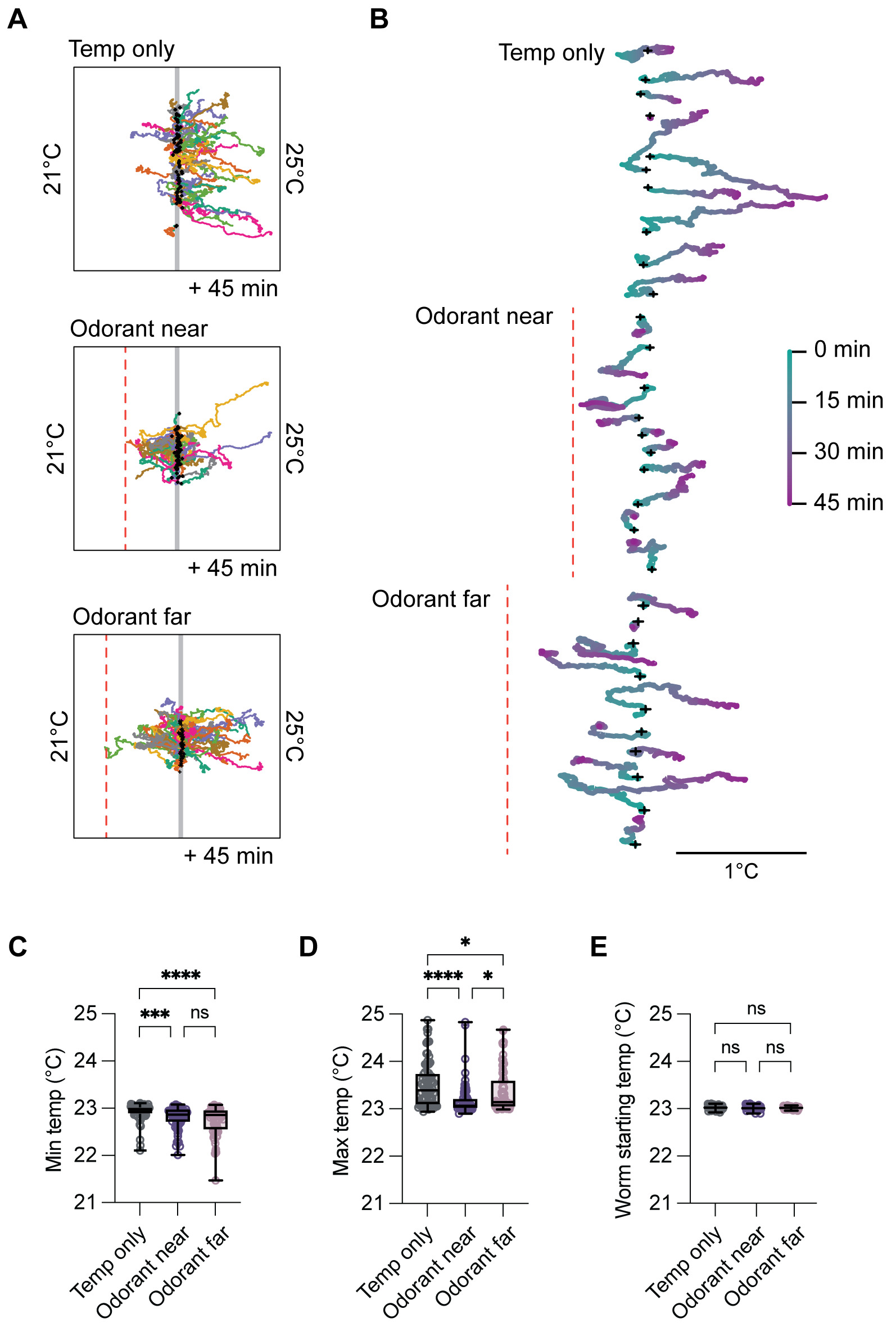

Supplement: S7 Fig — A) Tracks of worms in a ~21–25°C pure thermotaxis gradient or a thermal gradient with an attractive odorant (3m1b, pure) placed near (at 22.5°C) or far (at 22°C) from Tstart (23°C). Assay duration: 45 minutes. Colored tracks represent the path of individual worms. Black crosses represent the starting location of individual worms. The grey line represents Tstart = 23°C. B) Randomly selected representative tracks of individual S. stercoralis FLFs in each experimental condition from panel A. Tracks are color-coded by time. Vertical red hash lines represent the position of the odorant. Black crosses show the starting location of individual worms. C) Quantification of the minimum temperature experienced by S. stercoralis FLFs in a ~21–25°C gradient with and without 3m1b. The minimum temperature experienced in the presence of an odorant was significantly lower than in the temperature-only condition. Icons indicate responses of individual worms, boxes show medians and interquartile ranges, and whiskers show min and max values. ns = not significant, ***p<0.001, ****p<0.0001, Kruskal-Wallis test with Dunn’s multiple comparisons test. D) Quantification of the maximum temperature experienced by S. stercoralis FLFs in a ~21–25°C gradient with and without 3m1b. The maximum temperature experienced in the presence of an odorant was significantly lower than in the temperature-only condition. n = 76 worms for temperature only (7 assays over 5 days), n = 65 worms for odorant near (6 assays over 3 days), and n = 66 worms for odorant far (6 assays over 3 days). *p<0.05, ****p<0.0001, Kruskal-Wallis test with Dunn’s multiple comparisons test. E) Quantification of the starting temperature experienced by S. stercoralis FLFs in a ~21–25°C gradient with and without 3m1b. The starting temperatures of worms were not significantly different between the experimental conditions. ns = not significant, Kruskal-Wallis test with Dunn’s multiple comparisons test. (TIF) [file pntd.0012529.s007.tif]

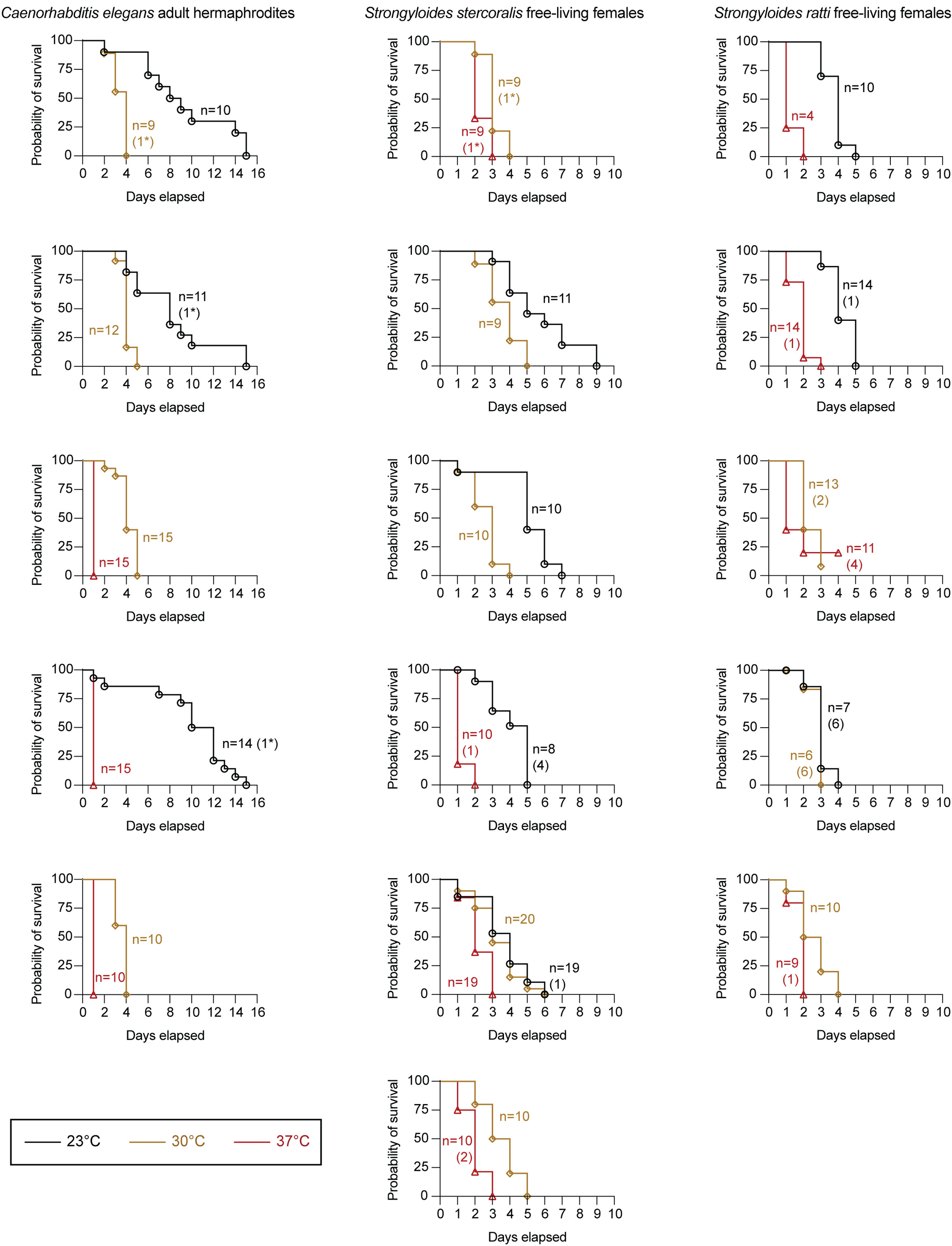

Supplement: S8 Fig — Probability of survival over time for C. elegans adult hermaphrodites, S. stercoralis FLFs, and S. ratti FLFs. Plots show survival curves for independent experiments. n = number of events (number of censored worms). When the number of censored worms is marked with an asterisk (*), the date of censoring is unknown, and the censored worms are excluded from plots and statistical analyses. (TIF) [file pntd.0012529.s008.tif]

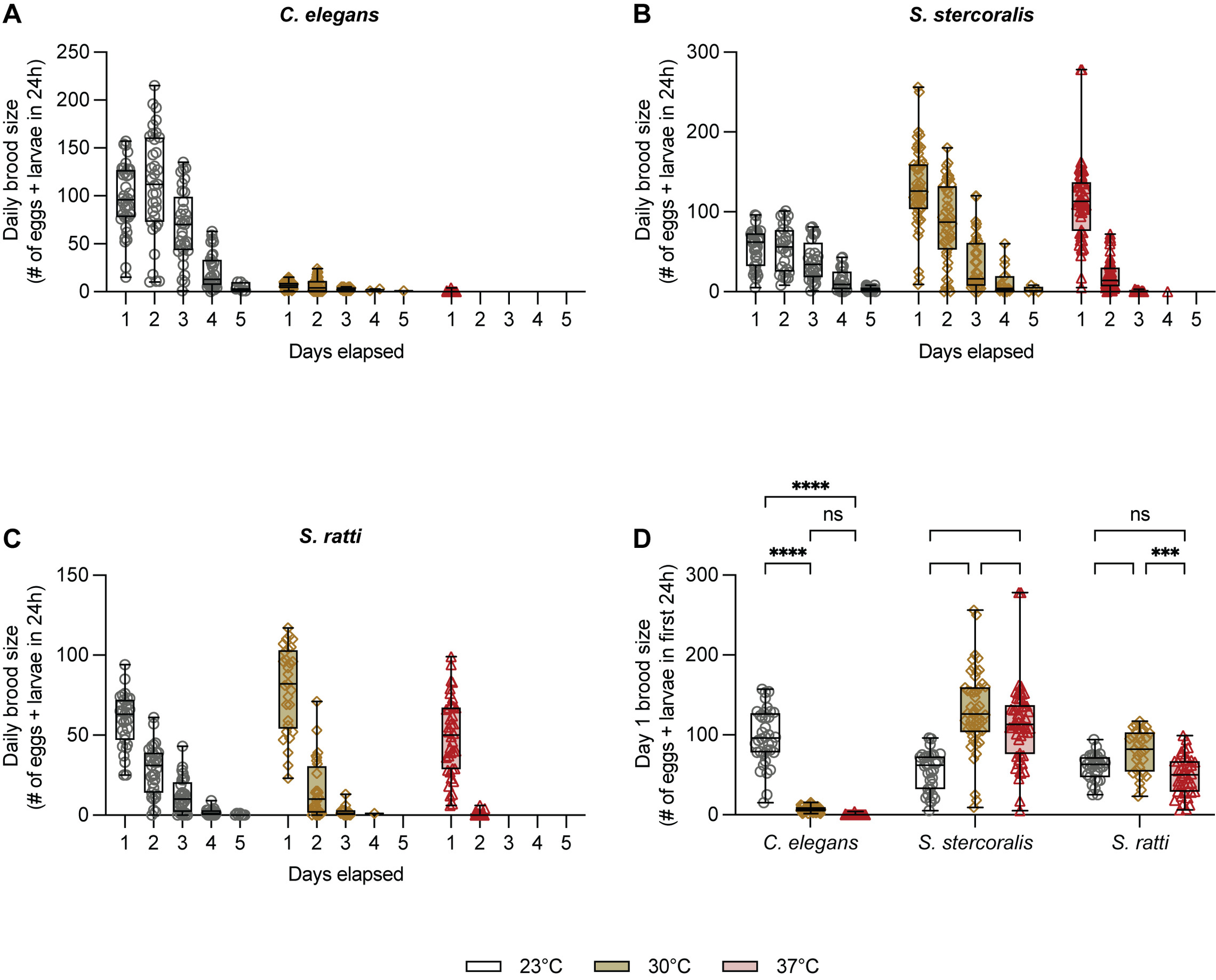

Supplement: S9 Fig — A-C) Brood size (eggs + larvae) recorded each day for C. elegans adults (A), S. stercoralis FLFs (B), and S. ratti FLFs (C) as a function of incubation temperature. Grey circles = 23°C; yellow diamonds = 30°C; red triangles = 37°C. Icons indicate brood sizes of individual worms, boxes show medians and interquartile ranges, and whiskers show min and max values. D) Day one brood size across species. S. stercoralis FLFs showed a significant increase in brood size on day 1 of the brood size assay at 30°C and 37°C. S. ratti FLFs showed a significant increase in brood size on day 1 of the brood size assay at only 30°C. In contrast, C. elegans adults showed a significant decrease in brood size on day 1. n = 31–54 adult worms. ns = not significant, ****p<0.0001, two-way ANOVA with Tukey’s multiple comparisons test. (TIF) [file pntd.0012529.s009.tif]
