# Supplementary material for: Thermosensory behaviors of the free-living life stages of Strongyloides species support parasitism in tropical environments
Source: PLoS Negl Trop Dis. 2024 Dec 17;18(12):e0012529. doi: 10.1371/journal.pntd.0012529 (PMC11687888; doi:10.1371/journal.pntd.0012529)
Supplement: Attachment — Submitted filename: Gregory et al 2024 response to reviewers.docx [file pntd.0012529.s011.docx]

**Response to Reviewer Comments:**

**Thermosensory behaviors of the free-living life stages of *Strongyloides* species support parasitism in tropical environments**

We appreciate the great enthusiasm of all three reviewers for our manuscript. In response to their insightful comments and suggestions, we have made changes to our manuscript as described below.

**Reviewer 1**

*“In this manuscript the authors compare the thermotactic behavior of the facultative free-living adults of two species of the nematode genus Strongyloides, which are small intestinal parasites and compare it with the corresponding behavior of the infective larvae of the same species and of adults of the non-parasitic model nematode C. elegans.  The study is interesting and well done. The manuscript is very well written and easy to follow. The rational and the results are presented in a well-understandable form. Below, I list a number of points, I recommend the authors to address prior to acceptance of this manuscript. Only one of these points is major.”*

1.1) “Although the authors do mention in the introduction that there are biological within species differences between different isolates, they interpret their results in this study always with respect to species. Given that for each species only one isolate (the respective laboratory isolate) was analyzed this results in an overinterpretation of the data. I do not think that it is clear if the differences observed between *S. stercoralis* and *S. ratti* are differences between species or between isolates. I am not asking for the inclusion of additional isolates but the authors should discuss more clearly that their data are valid for the particular isolates and that it is not known if similar differences might also exist between isolates of the same species.”

We agree that the distinction between isolates and species is unclear. We have added a sentence to the discussion that reemphasizes that our results are limited to a single isolate of either species:

“It is important to note, however, that our findings are limited to a single laboratory isolate for each *Strongyloides* species and differences in the thermotaxis behaviors and physiological characteristics between isolates of the same species may exist.”

1.2) “Fig. 1E (similar in Fig. S2D): It is indicated that there are a few *Strongyloides* with negative thermotaxis but in Fig. 1BC no such examples are shown. Although the authors do mention that they show representative (and not all) tracks, this is a bit confusing.”

We have added a new supplemental figure (Fig. S2) that shows all recorded tracks for both Figure 1 and Figure 2.

1.3) “Line 289: At this place it is not clear if the temperatures tested are indeed noxious for *Strongyloides*.”

We have replaced the sentence on line 289 with the following statement:

“Surprisingly, neither of the *Strongyloides* species displayed thermal preferences that matched those of *C. elegans* engaged in noxious temperature avoidance: *S. stercoralis* FLFs continued to show positive thermotaxis towards 34°C and *S. ratti* FLFs did not significantly disperse from their starting temperature of 30°C (Fig. 2B-E; Fig. S2B).”

1.4) “Line 291: Please comment if the [*S.ratti*] were still fully motile at 30°C.”

We agree that one possible explanation for the lack of dispersal by *S. ratti* FLFs is that exposure to 30°C impedes their motility, whereas exposure to 23°C (and the ~21-25°C gradient) does not. Our data indicate that this is not the case: the mean speed and total distance traveled by *S. ratti* FLFs is the same in both the ~21-34°C gradient and the 21-25°C gradient. Instead, we found that the trajectories of *S. ratti* FLF at 30°C were significantly more circuitous. We have added these quantifications as a new supplemental figure (Fig. S4). Additionally, we have added the following to our results section:

“The lack of dispersal by *S. ratti* FLFs is not due to reduced motility (quantified as mean speed and total distance traveled), but instead arises because their trajectories are more circuitous (Fig. S4).”

1.5) “Lines 309,313: It is a bit confusing that in the text the gradient is described as 12-22°C but in Fig. S5 it is labelled as 13-23°C. Similar small inconsistencies occur also at other places, for example between Fig. S6A and its legend.”

We have adjusted the gradient descriptions to remove all inconsistencies.

1.6) “Fig. S4 is not mentioned in the text”

We have removed Figure S4.

1.7) “Line 315: "Fig. S5D, F" (the 5 is missing).”

We have added the correct figure number.

1.8) “Fig 3B: I cannot really understand how to interpret the heatmap.”

We apologize for any confusion regarding the heatmap in Figure 3B. We have expanded the figure legend to help clarify our visualization of the worm trajectories. We also now direct readers to Fig. S6, which shows this data as worm track plots similar to Fig. 1A-C.

1.9) “Fig. 4: Is this figure really necessary?”

We generated Fig. 4 to schematize the complex sensory hierarchy that we have uncovered in our study, and to visualize our findings in the context of the thermosensory behaviors of *Strongyloides* infective larvae and *C. elegans*. We find Fig. 4 helpful for our own understanding, and hope that some readers will agree.

1.10) “Lines 619,620 (legend to Fig. 5): Please indicate here how many worms per experiment.”

We have added the number of worms for each temperature and species to the figure legend.

1.11) “Lines 675,676: Mention that autoinfection is specific for *S. stercoralis* and does not occur in *S. ratti.*”

We have changed the indicated sentence to specify that autoinfection only occurs in *S. stercoralis*: “In S. stercoralis, autoinfection can occur when eggs hatch within the host and develop into autoinfective larvae iL3s.”

In addition, we have retained the legend for Fig. S1B, which states that for *S. ratti,* “the stages of infection are similar to *S. stercoralis*, with the following differences: a) the progeny of parasitic females leave the host exclusively as unhatched eggs; **b)** **the lack of precocious hatching precludes autoinfection of original hosts.**

**Reviewer #2**

*“Gregory and colleagues present an elegant study of the thermal preferences and of Strongyloides free-living adult nematodes in comparison to the well-studied preferences of C. elegans. This work builds upon previous studies from the authors on the thermal and odorant preferences of infective-stage Strongyloides. The data presented demonstrate that Strongyloides FL adults are attracted to higher temperatures but that at these temperatures' lifespan is reduced. Interestingly at higher temperatures Strongyloides FL adults appear to compensate for reduced lifespan by increasing reproductive capacity; this is especially true for S. stercoralis which is more adapted to tropical environments in comparison to S. ratti which has a global distribution.*

*This manuscript not only provides comparative data for nematodes which occupy similar environmental niches (C. elegans and Strongyloides) but also begins to provide insight into the stage-specific behaviours of parasitic nematodes which facilitate their success. It also presents a novel dataset on which to build functional studies that can tease apart the underlying mechanisms responsible for such behaviours.*

*The broader impact of these findings on the understanding of parasite transmission and infection in the context of climate change and, the potential to leverage such knowledge in pursuit of novel forms of parasite control, is particularly significant. This is an exceptionally well written manuscript built upon well considered hypothesis and experiments and illustrated with informative figures that are presented beautifully. As such, I have no significant suggestions for improvement and congratulate the authors on their work. I am keen to see this study accepted for publication in its current form.”*

We sincerely thank Reviewer 2 for their kind comments on our manuscript.

**Reviewer #3:**

*“The study is very well written, results presented in efficient and beautiful figures and the overall conclusions are supported by the data. The results are of potential relevance for tropical disease understanding and certainly deserve being published as they are right now. I congratulate the author for this very interesting study. I only have minor concerns, not requiring additional experiments:”*

3.1) “I understand the choice of *C. elegans* as comparison point as it is very well studied. The classical N2 strain used here was originally isolated in Bristol, UK, and potentially further drifted genetically in the lab before being fixed as reference strain. It would have been interesting to compare *Strongyloides* with a free-living tropical species (*e.g.,* the relatively well-studied *C. briggsae*). Obviously, it is too late to change this. But the authors could at least discuss this point and make it more apparent that the *C. elegans* strain used as ‘reference’ free-living species also diverges quite a lot in term of habitat.”

To address this comment, we have added the following text to our discussion:

“Notably, we use the *C. elegans* N2 strain, which has likely adapted to maintenance in laboratory environments. Previous studies have found extensive variation in the lifetime fecundity and temperature preferences of geographically distinct *C. elegans* isolates, indicating that thermoregulatory strategies can vary even within a single species [46,48,75,76].”

3.2) “it would be fair to mention a previous study in *C. briggsae* doi:10.1242/jeb.075408. My understanding of this article is that tropical isolate of this species were performing robust negative thermotaxis. For example, JU1341 was isolated in Kerala, India, and behaved very similarly to N2. What’s more several tropical isolates displayed even more pronounced cryophilic drives, which is opposite to free-living *Strongyloides* in the present study, and which reinforces the idea that the positive thermotaxis (+ physiological adaptation to higher temperature for growth) in the parasitic species is really part of a move to favor future infectivity in the next generation.”

We agree that these findings are very relevant. We have added the following to our discussion (bold text indicates added text):

“Is the ability of *Strongyloides* post-parasitic eggs to maintain high hatching viability in warm temperatures a common feature of nematodes that are endemic to tropical and sub-tropical regions? **Tropical isolates of the free-living nematode *Caenorhabditis briggsae* are more cryophilic than even *C. elegans*, suggesting that preference for warm temperatures is not an intrinsic feature of nematodes found in tropical climates [49,83].**”

3.3) “Another limitation is that we don’t know to what extent the linear gradient assay in 2D will resemble what the worms are doing in the soil (complex 3D environment). Even if this limitation regards virtually every studied conducted with nematode in lab settings so far, it would be fair to the reader to brought it up explicitly.”

We have added the following to the discussion section:

“Furthermore, our experiments utilize 2D linear environments and stable environmental temperatures; exposure to more complex 3D environments that display diurnal fluctuations in temperatures could reveal more nuanced behaviors across both *C. elegans* and *Strongyloides* species.”
